# Proteolysis-targeting chimeras and their implications in breast cancer

**DOI:** 10.37349/etat.2021.00060

**Published:** 2021-12-31

**Authors:** Angeles C. Tecalco-Cruz, Jesús Zepeda-Cervantes, Josué O. Ramírez-Jarquín, Alberto Rojas-Ochoa

**Affiliations:** 1 Programa en Ciencias Genómicas, Universidad Autónoma de la Ciudad de México (UACM), CDMX, Mexico City 03100, Mexico; 2 Departamento de Microbiología e Inmunología, Facultad de Medicina Veterinaria y Zootecnia, Universidad Nacional Autónoma de México (UNAM), CDMX, Mexico City 04510, Mexico; 3 Instituto de Fisiología Celular, Universidad Nacional Autónoma de México (UNAM), CDMX, Mexico City 04500, Mexico; 4 Instituto Nacional de Pediatría (INP), CDMX, Mexico City 04530, Mexico; University of Southampton, UK

**Keywords:** Estrogen receptor alpha, breast cancer, degradation via the ubiquitin-proteasome system, proteolysis-targeting chimeras

## Abstract

Breast cancer (BC) is a highly heterogeneous neoplasm of the mammary tissue, causing the deaths of a large number of women worldwide. Nearly 70% and 20% of BC cases are estrogen receptor alpha positive (ERα+) and human epidermal growth factor receptor 2-positive (HER2+), respectively; therefore, ER and HER2 targeted therapies have been employed in BC treatment. However, resistance to these therapies has been reported, indicating a need for developing novel therapeutic strategies. Proteolysis-targeting chimeras (PROTACs) are new, promising therapeutic tools designed with a bimodular structure: one module allows specific binding to target proteins, and the other module allows efficient degradation of these target proteins. In this paper, PROTACs and their potential in controlling the progression of ERα and HER2+ BC are discussed.

## Introduction

The ubiquitin-proteasome system (UPS) refers to the ubiquitination of proteins followed by their degradation via the 26S proteasome. Ubiquitin (Ub) is an 8-kDa and 76-amino acid protein that is conserved from yeast to humans. Because of a conjugation motif on its C-terminus, Ub can form an isopeptide bond to specific lysine residues of several proteins. This process called ubiquitination occurs by three sequential reactions, requiring E1-Ub activating enzyme, E2-Ub conjugating enzyme, and E3-Ub ligase enzyme. The E3-Ub enzymes allow for a high degree of substrate specificity in ubiquitination [1–3].

Proteasome 26S is composed of catalytic core 20S and regulatory unit 19S. The 19S unit allows polyubiquitinated proteins to generate unfolded proteins that are then translocated to the 20S catalytic unit [4]. In summation, a polyubiquitinated protein is recognized by proteasome 26S, transported to the 20S core particle, and converted into oligopeptides by a variety of enzymes that promote oligopeptide release from the proteasome. Ub is recycled at the end of this process [5].

There are approximately 600 members of the E3-Ub ligase family, with really interesting new gene (RING) E3 ligases being the most common [1, 6–8] (Table 1). The bonds of Ub can form monoubiquitination of different residues on one target protein and polyubiquitination. Polyubiquitination with Ub chains occurs because of Ub lysine residues 6, 11, 27, 29, 33, 48, and 63, allowing for the possibility of branched Ub structures [9–12].

**Table 1.** E3-Ub ligases types

**Main types of E3 ligases**	**Characteristics**	**Action mode**
RING E3 ligases	Contains a RING domain or U-box domain	Ub is transferred in one step from the E2-conjugating enzyme to the substrate
Homologous to the HECT E3 ligases	Subfamilies: 1. RCC1-like domains; 2. tryptophan-tryptophan domains; 3. ligases lacking these domains	Ub is transferred in two steps: these ligases first transfer Ub from the E2-conjugating enzyme to themselves and subsequently to the substrate
RBR E3 ligases	Contain an IBR which separates two predicted RING domains (RING1 and RING2)	

RCC1: regulator of chromosome condensation 1; RBR: RING-in-between-RING; HECT: E6AP carboxyl terminus; IBR: in between ring

The ubiquitination process is modulated through deubiquitinases (DUBs) that reverse ubiquitination, and almost 100 DUBs are known in humans [9–11]. Furthermore, Ub receptors are Ub-binding domains that recognize the Ub mark from proteins modified by ubiquitination, and this interaction can impact the function or signaling of these proteins [5, 13–16]. Although polyubiquitination is classically associated with proteolytic activities, it is important to note that non-proteolytic functions can also be performed as a result of polyubiquitination in specific cases [17–21]. Additionally, monoubiquitination has been described as a modification implemented in the regulation of protein stability, transcription, and DNA repair.

In recent years, there has been an increasing number of studies on the deregulation of Ub-associated pathways and the functional implications of ubiquitination in many diseases, including cancer. Much of the knowledge about ubiquitination and the UPS is being utilized to generate new technologies that are useful as therapeutic strategies, such as proteolysis-targeting chimeras (PROTACs). We discuss PROTACs in the context of breast cancer (BC) in this review.

## PROTACs

**Figure 1.**
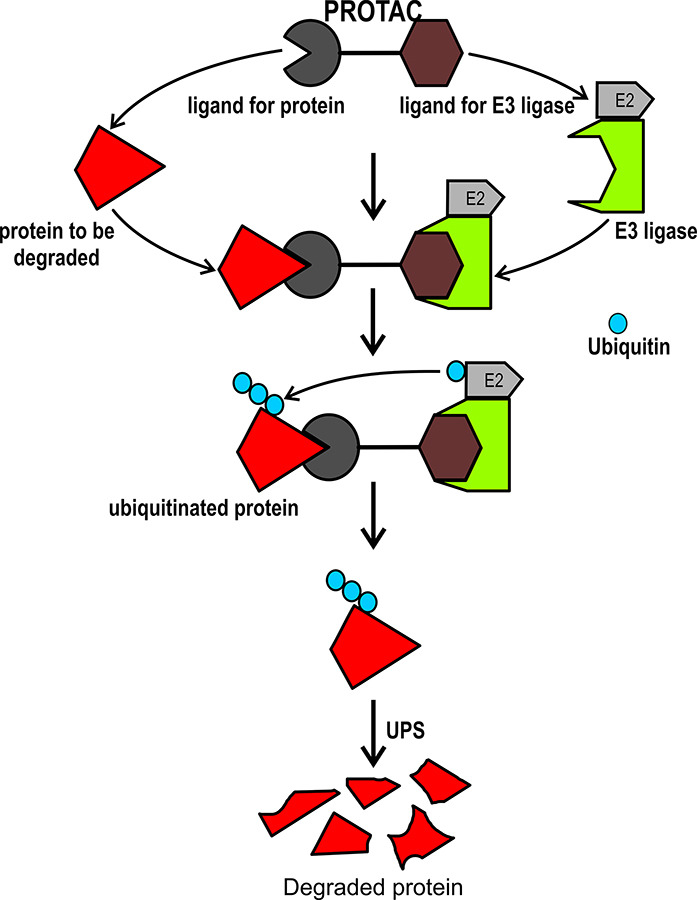
PROTACs. Representation of molecular mechanism that is used by PROTACs

PROTACs have a bifunctional structure designed to initiate the degradation of specific proteins through UPS. The bifunctional structure of PROTACs contains a module with the ability to bind to the target protein (core region) and another module responsible for recruiting an E3-Ub ligase (degron), both of which are linked to the target protein by a spacer [22]. The E3-Ub ligase marks the target protein for degradation, reducing the abundance and functionality of the target protein (Figure 1) [23, 24].

Domains and motifs required for interaction with E3-Ub ligases need to be considered in the construction of PROTACs. The large peptides which were initially used in PROTACS were not completely functional due to their limited penetration into the cell [23]. In one of the first studies, PROTACs containing phosphopeptide IkappaB (IκB) have been shown to recruit the SCF^β-TrCP^ Ub ligase complex, subsequently promoting the degradation of methionine aminopeptidase 2 (MetAP-2), which has been linked to cancer [23, 24]. To date, several E3-Ub ligases have been considered in the design of PROTACs, some of which are shown in Table 2 [23, 25–27].

**Table 2.** Some E3-ligases used for PROTACs

**Abbreviation**	**Name**
MDM2	Murine double minute 2
CRBN	Cereblon
VHL	Von Hippel-Lindau tumor suppressor
IAPs	Inhibitor of apoptosis proteins

PROTAC technology has advantages over other treatments. PROTACs directly degrades the target protein, even undruggable targets, since PROTACs design allows the binding to any site of the target protein without requiring a high affinity. Moreover, resistance to conventional treatments for cancer is common, whereas the development of resistance to PROTACs is not feasible. Thus, PROTACs degrade overexpressed or mutated proteins associated with resistance to drugs. In addition, PROTACs have high potency, efficacy, and less toxicity [28].

Although PROTACs are a promising means of inducing the degradation of specific target proteins, they also have disadvantages. Future PROTAC studies could investigate improvements regarding molecule size, pharmacokinetics, and administration. Since PROTACs are designed to bind to specific targets, sometimes the large size of molecules can affect their bioavailability and solubility. PROTAC bioavailability is also affected by intraperitoneal and subcutaneous administration which are the two current standards of administration; accordingly, new intravenous and oral methods of administration are being investigated [22, 29–31].

## BC: luminal and human epidermal growth factor receptor 2-overexpressing

BC is one of the main causes of death among women worldwide. There is high molecular heterogeneity in mammary tumors, indicating the molecular complexity that influences BC’s classification, biomarker determination, and pharmacological treatments (Table 3) [32–34].

**Table 3.** BC types and therapies

**BC type**	**Biomarkers**	**Therapy**
Luminal A-like	ERα+, PR ≥ 20%, HER2-, Ki67 < 20%	Endocrine therapy
Luminal B-like	ERα+, PR < 20% and/or HER2+ and/or Ki67 ≥ 20%	Endocrine therapy
HER2-overexpression	ERα-, PR-, HER2+	HER2-targeted therapy
Basal-like	ERα-, PR-, HER2- (triple-negative)	Not responsive to endocrine or anti-HER2 therapies

ERα+: estrogen receptor alpha positive; PR: progesterone receptor; HER2-: human epidermal growth factor receptor 2-negative; HER2+: HER2-positive; Ki67: marker of proliferation Ki-67

### ERα+ BC

**Figure 2.**
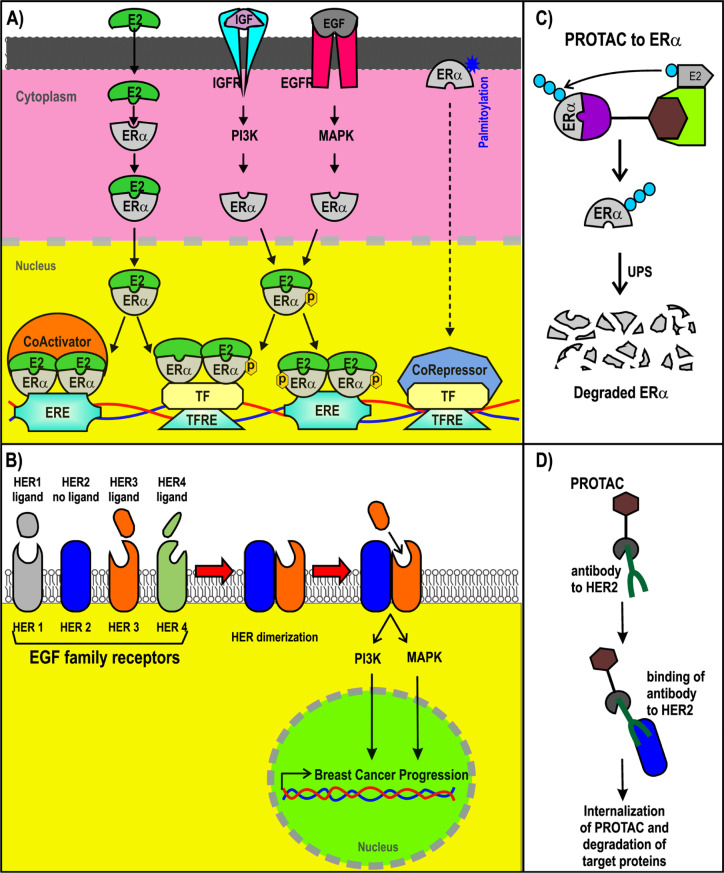
ER+ and HER2+ BC and PROTACs. A) Signaling pathways triggered by ER; B) representation of PROTACs designed for ER; C) signaling pathways of HER2+; D) design of antibody (Ab)-PROTAC conjugated using an Ab for HER2. ERE: estrogen response element; IGFR: IGF receptor; PI3K: phosphatidylinositol-4,5-bisphosphate 3-kinase; EGFR: EGF receptor; MAPK: mitogen-activated protein kinase; TF: transcription factor; TFRE: TF response elements; ER+: ER-positive

Most BC cases (~70%) are luminal type and thus are ERα+. ERα is a nuclear receptor activated by estrogens. It is also activated via phosphorylation induced by growth factors such as insulin-like growth factor (IGF) and epidermal growth factor (EGF) in BC cells [32, 33, 35, 36]. Although ERα is a nuclear receptor, it is also associated with cytoplasmic proteins and transmembrane receptors. ERα can also be membrane-associated via its palmitoylation [37, 38]. Extranuclear ERα is part of the signaling route that may lead to gene regulation [39–41]. Nuclear ERα can act as a transcription factor or a coregulator that modulates gene expression [42, 43]. Estradiol, the most abundant estrogen, induces the expression of its target genes through ERα activation. Many of those target genes are associated with pro-tumor activity in BC cells (Figure 2) [44, 45].

The structure of ERα, from the N-terminus and C-terminus, consists of the activation function domain-1 (AF-1), the DNA-binding domain (DBD), a hinge region, the ligand-binding domain (LBD), and the activation function domain-2 (AF-2) [46, 47]. AF-2 recruits coregulators in an estradiol-dependent manner. AF-1 does the same in an estradiol-independent manner. AF-1 (in residues that include S118 and S167) is a target of phosphorylation by EGF and IGF signaling, activating the action of the receptor [44, 48–51]. The expression and recruitment of coactivators for ERα are usually increased in BC, enhancing the expression of their target genes [30, 45, 52–54]. Through DBD, ERα binds to EREs contained in the enhancers and promoters of their target genes to modulate its expression. Moreover, forkhead box A1 (FOXA1) and GATA binding protein 3 (GATA3) are considered pioneer factors that facilitate the recruitment of ERα to ERE in DNA regulatory sequences [55, 56].

### HER2-overexpression in BC cells

Approximately 20% of all BCs are HER2+ [57]. HER2 is a member of the EGF receptor family and is also known as the proto-oncogene neu and ErbB2. HER2+ BC cases are characterized as being highly aggressive, recurrent, and having a poor prognosis. Nearly 50% of these cancer cases also display co-expression with hormone receptors [58, 59]. The gene that encodes HER2 is on chromosome 17q12 and is known as ErbB2 receptor tyrosine kinase 2 (*ERBB2*) [60, 61]. HER2 is a transmembrane receptor with tyrosine-protein kinase activity, but it does not have the ability to recognize growth factors nor can it be activated by growth factor ligands directly, as it lacks the ligand-binding extracellular domain [60, 62, 63]. HER2 associates with other members of the EGF receptor family that do possess the ligand-binding ability, forming heterodimers that trigger downstream signaling, such as phosphatidylinositol-3 kinase and MAPK activation, promoting BC progression [60, 62].

## ERα and HER2-targeted therapies for BC

### Endocrine therapies

Endocrine therapies (ET) are used for ERα+ BC cases because they enclose aromatase inhibitors (AIs) and selective estrogen receptor modulators (SERMs). AIs block estradiol production, whereas SERMs are a type of ET that acts by competing with estradiol by binding to ERα and inhibiting the recruitment of coactivators. SERMs promote the interaction with corepressors to avoid the transcription of estradiol-target genes in BC cells [64–66]. A common problem of these types of ET is *de novo* resistance and acquired resistance [65–69]. The mechanisms underlying the resistance to ET are not completely understood, but they have been shown to be related to signaling pathways activated by ERα [69, 70].

Interestingly, mutations in estrogen receptor 1 (*ESR1*), the gene that encodes ERα, specifically the mutations Y537S, Y537N, Y537C, D538G, and E380Q, have been found in circulating tumor DNA and biopsies of metastatic BC with resistance to ET [71–73]. These mutations encompass the LBD and allow ERα to act in an estradiol-independent manner, modifying the profile of ERα target genes, some of them associated with endocrine resistance to ET [72, 74–77].

Selective estrogen receptor degraders (SERDs) are another ET that are considered as antiestrogens and antagonize estradiol [66–68]. However, the binding of SERDs to ERα promotes the polyubiquitination and degradation of this receptor through the UPS [64, 78, 79]. To date, fulvestrant is the most important SERD described since even the commonly reported *ESR1* mutations seem to be sensitive to this SERD [72, 75, 76]. Although several SERDs are being evaluated, fulvestrant is the first SERD approved as a first-line ET to date [80]. It is hoped that the new generation of SERDs will have improved bio-dispensability and administration routes in comparison with fulvestrant, which is administrated intramuscularly and has a limited bio-dispensability [81–86].

The mechanism of SERDs is based on ERα degradation via the UPS, which has significant advantages compared to the mechanisms of SERMs. New therapeutic strategies that are also based on ERα polyubiquitination as a marker for its degradation via the UPS are PROTACs (Figure 2C), as will be discussed in the latter half of this review.

### HER2-targeted therapies

An improvement in the prognosis of HER2+ BC has been observed as a result of different therapeutic strategies. The first approved therapy for HER2+ BC was adjuvant chemotherapy called trastuzumab, which consists of a humanized monoclonal Ab that specifically recognizes HER2, inhibiting HER2 dimerization [87]. Other HER2-directed monoclonal antibodies, such as pertuzumab, have now been generated. Additionally, novel strategies have emerged, including Ab-drug conjugates (ADCs) and tyrosine kinase inhibitors (TKIs) [88].

ADCs are recombinant monoclonal antibodies covalently bonded to a cytotoxin, generating a chemotherapeutic drug specifically targeted against HER2. This allows for intracellular delivery of the cytotoxic drug [89]. The bonds between the monoclonal Ab and cytotoxic drug are cleaved once the Ab binds to its target antigen, allowing the cytotoxic drug for use on other cells [89, 90]. Trastuzumab emtansine (T-DM1) is a non-cleavable ADC, showing antitumor activity in HER2+ BC [91]. Despite its therapeutic effects, since HER2 can be expressed in other cells and since cytotoxic drugs can be internalized in cells with low HER2 expression, gastrointestinal, hematologic, and hepatic toxicity has been reported. Therefore, further investigations are required to ensure the safe use of the ADCs in BC [92, 93].

TKIs, such as lapatinib, neratinib, tucatinib, and pyrotinib, are also used to treat HER2+ BC. TKIs can be used in combination with chemotherapy [88]. TKIs are of interest for the treatment of HER2+ cells that are resistant to the other therapies. The resistance to therapies has been associated with HER2 reactivation, HER2-dependent downstream signaling, upregulation of the HER receptor family, and HER2 mutations [94–97]. For example, L755S is an acquired activating mutation of HER2 identified in HER2+ metastatic BC, which may prevent the binding of the monoclonal antibodies and lapatinib activity [94, 95, 98]. With the use of TKIs as a BC treatment even with HER2 mutations, there is an increasing interest in developing novel TKIs for HER2+ BC [94, 96].

## PROTAC for BC

### PROTACs for ERα

**Figure 3.**
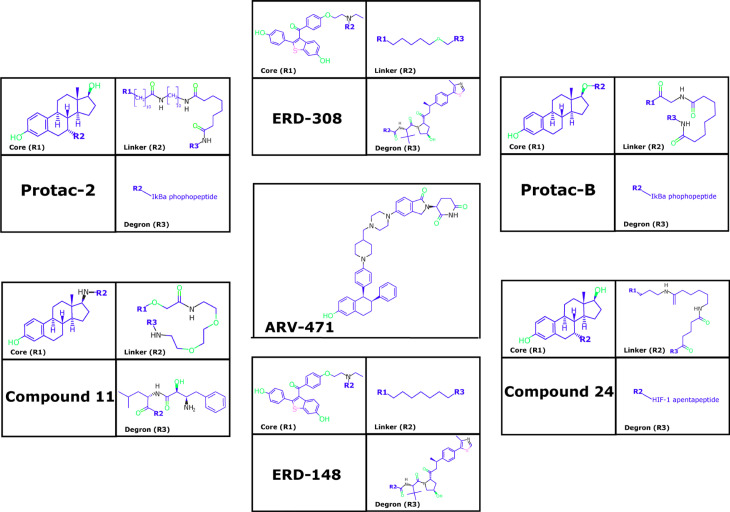
Representative PROTACs for ER

PROTAC-2, PROTAC-B, compound 24, compound 11, and estrogen receptor degrader (ERD)-148, among other PROTACs, have been designed to induce the reduction of ERα levels in BC cells (Figure 3) [99–101]. PROTAC-2 was designed with an estradiol molecule to bind to ERα and the phosphopeptide of IκBα that recruits SCF^β-trcp^ Ub ligase complex, leading to ERα degradation [102]. PROTAC-B displays a peptide from hypoxia-inducible factor-1α (HIF-1α) that allows for the recruitment of the E3-Ub ligase VHL [103, 104]. E2-octa and E2-penta contain synthetic peptides derived from HIF-1α, resulting in an efficient reduction of ERα levels [101]. Additionally, specific and non-genetic IAP-dependent protein eraser (SNIPER) is a PROTAC formed by a derivative of estradiol along with a bestatin amide that binds the cellular inhibitor of apoptosis protein 1 (cIAP1) ligand which induces ERα degradation [23, 105, 106].

Another example is compound 1–6, which was designed as a peptide-based PROTAC with better penetration [26]. Similarly, TD-PROTAC is another peptide-based PROTAC constructed from TD-PERM (a stabilized peptide that binds to ERα), the linker, and the hydroxyproline-containing pentapeptide from HIF-1α to recruit the VHL E3 Ub ligase complex. TD-PERM promotes 75% reduction in tumor volume in michigan cancer foundation-7 (MCF-7) mouse xenograft model [99].

Furthermore, ERD-308 PROTAC induces ERα degradation by reducing cell proliferation to a greater extent than fulvestrant (> 95% of ERa degradation at concentrations as low as 5 nmol/L in MCF-7 and T47D BC cells) [25]. ARV-471 PROTAC reduces ERα levels and its Y537S and D538G variants. Furthermore, ARV-471 significantly reduces estradiol-dependent MCF-7 xenograft tumors and tumor ER protein levels (> 90%) [27, 107]. Other PROTACs have also been evaluated *in vitro* as well as using *in vivo* models, and indicated a reduction in cell proliferation, inhibition of estradiol target genes, and induction of apoptosis in both models. This suggests that PROTACs have a strong antitumor therapeutic effect [99]. However, ARV-471 has been proposed as the best-in-class oral ERa PROTAC due to its results in preclinical trials. Currently, ARV-471 is in phase 2 studies to treat advanced or metastatic ER+/HER2+ BC [107, 108].

### PROTACs in HER2+ BC

New PROTAC designs consider the use of monoclonal antibodies to target specific proteins. For example, a study generated a new PROTAC with Ab linker named Ab-PROTAC3, a trastuzumab-PROTAC conjugate. This conjugate has the ability of PROTACs to recruit E3-Ub ligase specifically to HER2+ cells through trastuzumab. Then, trastuzumab-PROTAC can be internalized into the cell, and active PROTAC is released to promote the degradation of bromodomain-containing protein 4 (BRD4) [109].

### PROTACs for other proteins in BC

PROTACs can be applied to other proteins implicated in the progression of BC. For example, B-cell lymphoma-extra large (Bcl-xL) is overexpressed in several cancer types, including BC. The PROTAC named 8a can promote the Bcl-xL degradation in BC cells [110]. Additionally, the bromo and extra terminal domain proteins (BET) such as BRD2, BRD3, and BRD4 are epigenetic regulators considered potential targets for BC treatment. It has been generated PROTACs for BRD4 using VHL E3 ligase and for BRD2, BRD3, and BRD4, using E3 Ub ligase cereblon (CRBN), showing results in triple-negative BC [111, 112]. Thus, several proteins involved in BC may be targeted by PROTACs.

## Conclusions

Most of the BC cases are ERα+ and use endocrine therapy, but a common problem is resistance to these therapies. SERDs exhibit better therapeutic results with few instances of resistance compared to SERMs and AI. However, to date, fulvestrant is the only approved SERD. New SERDs are being evaluated, focusing specifically on improving bioavailability and administration routes [66]. PROTACs have emerged as promising therapies. Because their mechanisms are also linked to the UPS, it is hypothesized that PROTACs will be specific and efficient, without drug-resistance problems.

PROTACs require the definition of new “degron” motifs to have more recognition regions for their use [100]. This is important since PROTACs have demonstrated effective inhibition of ERα target genes and proliferation. However, further investigations are necessary to improve their cell penetration ability [99, 113–115]. PROTAC technology is associated with other therapeutic strategies, such as SERD-like PROTACs or Ab-PROTACs. It is expected that these molecules will show a higher specificity and adequate bioavailability and administration route than other therapies. Complete inhibition of tumor progression by new PROTACs is also expected. Moreover, it is relevant to consider that several mechanisms inhibit ERα degradation in BC cells, many of which are based on monoubiquitination by E3-Ub ligases and other cases by the actions of the DUBs [116]. Therefore, ERα stability in BC must be considered in future applications and designs of therapies based on the UPS.

Moreover, antibodies cannot be used for intracellular targets, requiring new PROTACs with good ability to penetrate cell membranes and excellent stability. Interestingly, an Ab-PROTAC has been designed in which trastuzumab Ab is used to recognize HER2. Through this specific system, the PROTACs are directed to the site where it has to induce degradation. These models indicate the versatility of PROTACs and the many ways that they can be studied to generate better degradation strategies for several targets. The current PROTACs could be improved with the fusion of peptides to penetrate cell membranes. More PROTACs targeting intracellular components related to BC with improved biochemical characteristics will need to be discovered and fully characterized. Thus, there is a clear need to find novel safe, and efficient drugs in treating BC. A central challenge going forward in this field will be to improve the pharmacokinetic properties of PROTACs.

In conclusion, PROTAC technology is being evaluated for its application in ERα+ and HER2+ BC treatments with promising results since PROTACs reduce the pro-tumor effects. Further investigation and improvements of these drugs are still required before PROTACs can be used as part of a new generation of drugs for the treatment of BC.

## Abbreviations

Ab: antibody
ADCs: antibody-drug conjugates
AF-1: activation function domain-1
BC: breast cancer
BRD4: bromodomain-containing protein 4
DUBs: deubiquitinases
EGF: epidermal growth factor
ER+: estrogen receptor-positive
ERE: estrogen response element
ERα+: estrogen receptor alpha positive
ET: endocrine therapies
HER2-: human epidermal growth factor receptor 2-negative
HER2+: human epidermal growth factor receptor 2-positive
HIF-1α: hypoxia-inducible factor-1α
IGF: insulin-like growth factor
MCF-7: michigan cancer foundation-7
PR: progesterone receptor
PROTACs: proteolysis-targeting chimeras
RING: really interesting new gene
SERDs: selective estrogen receptor degraders
SERMs: selective estrogen receptor modulators
TKIs: tyrosine kinase inhibitors
Ub: ubiquitin
UPS: ubiquitin-proteasome system
VHL: von Hippel-Lindau tumor suppressor

## Declarations

### Copyright

© The Author(s) 2021.
